# K-rice: a comprehensive database of Korean rice germplasm variants

**DOI:** 10.3389/fgene.2025.1544652

**Published:** 2025-06-05

**Authors:** Jeong-Gu Kim, Gyu-Hwang Park, Jinhyun Kim, Jinho Jeong, Tae-Ho Lee

**Affiliations:** Genomics Division, Department of Agricultural Biotechnology, National Institute of Agricultural Sciences, Rural Development Administration, Wanju, Republic of Korea

**Keywords:** rice, database, elite lines, Korean rice population, *Oryza sativa*

## Introduction

Rice cultivation in South Korea has a rich and extensive historical background, dating back to the Bronze Age (Jeong et al., 2021). Throughout history, rice has experienced various transformations in its status, from being a luxury food item to being used as animal feed owing to overproduction and reduced consumption. Recently, rice consumption in South Korea has declined owing to cultural changes and governmental policies. Consequently, the South Korean government is actively promoting rice consumption and focusing on rice exports to meet the global demand and supply chain requirements. The aroma of rice varies around the world, and South Korean rice varieties are primarily glossy, soft, and sticky rice, compared to other parts of the world. Additionally, factors such as enhancing the population-specific nutrients in rice, similar to the golden rice project (Wu et al., 2021), and reducing inputs for rice cultivation (Adu et al., 2022), which benefits the environment, the project such as Green Super Rice (Yu et al., 2022), are essential. Therefore, it is crucial to develop new rice varieties that cater to consumer preferences, combine traditional colored rice varieties, and enhance their nutritional value. Furthermore, the current scenario of rice overproduction is not stable, as Korean rice varieties have faced significant production losses due to temperature fluctuations in the past, such as a 17% loss in 1971 and 20% loss in 2003, as well as an 80% loss due to cold temperatures in 1980 (Jeong, et al., 2021). In light of the potential threat posed by current climate fluctuations to South Korea’s rice self-sufficiency, proactive measures must be taken to ensure continued rice production. Given the consequences of global warming and population expansion, which have resulted in a scarcity of rice worldwide, it is imperative to adopt proactive measures to promote sustainable rice production. To alleviate the strain on global food supply, the South Korean government is exploring the cultivation of non-japonica rice for export, which could also enable South Korea to establish itself as a formidable player in the global rice trade (Jeong, et al., 2021).

Considering rice breeding and its associated challenges, South Korea has been dedicated to extensive research and varietal improvements since the 1970s. This dedication began with the development of the high-yield cultivar “Tong-il,” which served as the foundation for the South Korean Green Revolution and allowed the country to achieve self-sufficiency (Kim et al., 2014). Over time, the emphasis has shifted from high-yield components to grain quality and stress resistance, resulting in the diversification of rice varieties. In total, 206 Korean rice varieties have been studied, among which a significant proportion has been categorized as good for consumption (Cho et al., 2009). It is noteworthy that the genetic diversity within these varieties is somewhat limited, with a large percentage originating from a few Korean-bred and Japanese stocks, which may pose challenges for future breeding owing to the potential reduction in hybrid vigor. In terms of disease resistance, a study of Korean rice varieties revealed the presence of major blast resistance genes, with a significant number of varieties containing genes that have origins in both Korean and Japanese japonica rice genotypes. The presence of these resistance genes contributes to the overall resistance of Korean rice to blast disease caused by *Magnaporthe oryzae* (Cho, et al., 2009). Although the presence of resistance gene pools ensures blast resistance, further breeding programs may be needed to address the genetic diversity issue to ensure the sustainable development of rice varieties in Korea (Cho, et al., 2009).

Despite significant progress in exploring the genetics of the rice genome using the available rice germplasm globally, a comprehensive analysis of 3,010 rice germplasms from diverse regions worldwide has been conducted as part of this endeavor (Wang et al., 2018). Additionally, contemporary rice breeding programs primarily incorporate genome sequencing to acquire detailed genetic knowledge and establish precision breeding to achieve desired outcomes (Paul, 2020; Wing et al., 2018). Furthermore, the era of genomics has facilitated researchers to pan-genome, which involves incorporating various cultivars and varieties to better comprehend genetics and biological functions associated with specific or multiple traits (Schreiber et al., 2024). Recently, the pan-genome of rice has shed light on the subpopulation structure of Asian rice varieties compared to the wild type (Zhou et al., 2023). The collective effort of the International Rice Genome Sequencing Project (IRGSP) consortium has resulted in a high-quality reference genome for rice *Oryza sativa* subsp. japonica cv Nipponbare (IRGSP v.1.0) (Kawahara et al., 2013), which offers comprehensive genomic information for *O. sativa*, a model species for monocotyledonous plants. This reference and its annotations are distributed with various additional assessments through primary rice databases such as RAP-db (https://rapdb.dna.affrc.go.jp), RGAP (http://rice.uga.edu), and Gramene (https://www.gramene.org). Additionally, insights into the rice pan-genome datasets can be obtained through RPAN (https://cgm.sjtu.edu.cn/3kricedb/index.php) and Rice RC (http://ricerc.sicau.edu.cn/RiceRC). Furthermore, the comprehensive catalogue of rice genes is organized at Rice Gene Index (RGI), and its variants are organized in databases such as Rice SNP-Seek, RPAN, and RiceVarMap (Yu et al., 2023). However, nationwide germplasm databases are the most valuable assets for researchers focusing on domestic varieties that are being improved. To facilitate this for Korean rice researchers, genome re-sequencing of the Korean rice population was conducted to identify effective breeding signatures for the green super rice strategy. This strategy aims to improve the Korean breeding efficiency for various associated issues. Despite the inclusion of 35 rice varieties from South Korea in the 3 K rice genome-resequencing project, additional elite lines were incorporated into the genome-sequencing process. The K-Rice database provides a comprehensive catalog of the elite Korean rice population and wild genomes, which will enable researchers to better understand the genetic variants present in these genomes and to identify breeding signatures primarily from Korean rice populations.

### Value of the data

The dataset presented in this study constitutes a useful and informative resource for understanding the genetic diversity of the Korean rice population. This dataset may prove to be a valuable asset for rice breeders and researchers, enabling them to conduct research on Korean rice varieties and develop new varieties that can address the challenges posed by the ongoing global warming crisis and the impending population increase.

## Materials and methods

### Collection of rice germplasm and phenotypic data

A total of 105 rice germplasms (85 elite cultivars and 20 wild accessions, detailed in Supplementary Table 1) were procured from the National Institute of Crop and Food Science (NICS), RDA, Korea. Along with the germplasm, NICS provided data for 15 phenotypic traits that they had previously investigated for these lines. These traits include protein content, milling recovery ratio, grain filling ratio, taste evaluation, head rice ratio, grain number, height, yield, panicle length, panicle number, grain length/width ratio, 1000-grain weight of brown rice, heading ecotype, blast resistance, and RSV (rice stripe virus) resistance. This phenotypic data is accessible within the K-RICE database for each corresponding germplasm.

### DNA sequencing and variant calling

Total DNA was isolated from the samples individually according to standard sequencing protocols. DNA was prepared using a TruSeq Nano DNA Prep Kit for Illumina sequencing. Each isolated DNA sample was sequenced using Novaseq6000 (Illumina), which is a short-read sequencing technique. The experiment was performed by Macrogen, an authorized service provider in South Korea. Illumina paired-end sequences were subjected to quality and adapter trimming using BBDuk v28.26. The processed reads were mapped to the *O. sativa* subsp. japonica cv Nipponbare (IRGSP v1.0) reference genome (Kawahara, et al., 2013) using Bowtie2 v.2.2.5(Langmead and Salzberg, 2012), and variant calling was performed with the Haplotype caller in the Genome Analysis Toolkit (GATK v4.2.0.0) (McKenna et al., 2010). SNPs were selected using GATK parameters, that is, a normalized quality score ≥2 and mapping quality ≥40. The SNPs were annotated using SnpEff v.4.2 (Cingolani et al., 2012).

### Establish database framework

**FIGURE 1:**
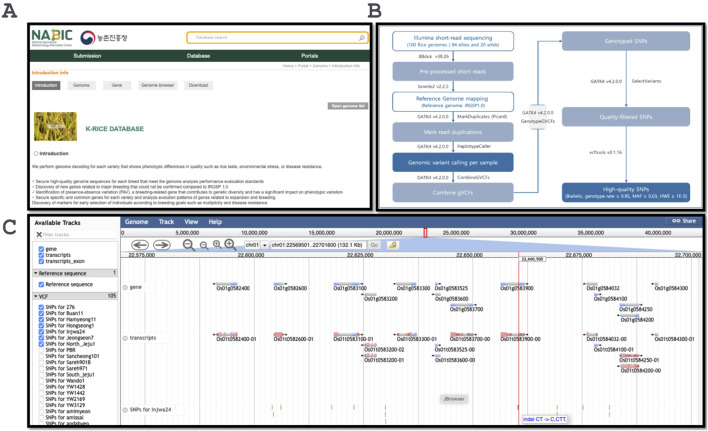
The design and output example of the database. **(A)** Index page of the K-Rice database; **(B)** The variants calling pipeline overview; **(C)** Genome browser with all 100 germplasm variant datasets.

The entire database of webpages was encoded using Java, and the database was accessed via the URL (http://nabic.rda.go.kr/post_jbrowse.do?data=K_RICE). The database was designed to facilitate effective exploration of the variant region using the genome JBrowse for all 100 VCF file tracks, along with the reference genome, which includes the transcripts, gene, and exon regions of the genes. The respective VCF files were also available for downloading (Figure 1).

### Preliminary analysis report

The overall quantity of sequence data generated from 105 rice germplasm samples (including 85 elite and 20 wild samples) using whole-genome short-read sequencing. Each sample generated approximately 11.9 GB of raw reads, which became 11.6 GB after processing. Of the processed reads, 98.2% were successfully mapped to the rice genome, with the mapped reads covering the genome being approximately 26.6-fold. The dataset also provided 15 phenotypic values, such as protein content, milling recovery ratio, grain filling ratio, taste evaluation, rice head ratio, grain number, height, yield, panicle length, panicle number, grain length/width ratio, 1,000 grain weight of brown rice, heading ecotype, blast resistance, RSV (rice stripe virus) resistance, to evaluate individual trait vigor (Supplementary Table 1). Moreover, the genomes were categorized into three primary groups based on their resistance to blast, striped leaf blight, and flowering time, as illustrated in Table 1, and shown to represent the coverage of the Korean germplasm.

**TABLE 1:** Overview of rice genome trait features in the K-rice database.

Traits	Level/Time	Japonica	Tongil-type
Resistance to blast	Weak	10	1
	Medium Weak	3	0
	Medium	13	3
	Medium Strong	3	2
	Strong	22	19
Resistance to Striped leaf blight	Weak	23	1
	Medium Weak	0	0
	Medium	1	2
	Medium Strong	1	0
	Strong	27	19
Flowering Time	Very early maturing variety	2	0
	Semi-early maturing variety	3	0
	Early maturing variety	14	1
	Medium maturing variety	16	15
	Medium late maturing variety	20	8
	Late maturing variety	0	3

Recognizing the importance of nationwide germplasm variation in rice, it is crucial for researchers to conduct extensive research and contribute to the diversification of rice varieties. The Korean National Agricultural Biotechnology Information Center, which formerly maintained the Korean rice germplasm, has organized the genetic variant data into the K-Rice database, which is now accessible to the public.

## Data Availability

The complete sequences generated in this study were deposited in NCBI Project accession no. PRJNA1180626.
